# A Challenge in Diagnosis of Cerebellar Hemangioblastoma

**DOI:** 10.7759/cureus.21713

**Published:** 2022-01-29

**Authors:** Ana Lopes dos Santos, Sara Trevas, Maria Luiza Rosado

**Affiliations:** 1 Internal Medicine Department, Hospital São Francisco Xavier, Lisboa, PRT; 2 Neurology Department, Hospital Pêro da Covilhã, Covilhã, PRT

**Keywords:** benign neoplasm, central nervous system neoplasm, von hippel-lindau disease, sporadic hemangioblastoma, hemangioblastoma

## Abstract

Hemangioblastomas are benign neoplasms, which are highly vascularized and have a slow-growing rate that typically affect the central nervous system; they account for about 1-2.5% of all intracranial tumors and for approximately 2-3% of all intramedullary neoplasms. We present a clinical case of cerebellar hemangioblastoma with six years of evolution, which illustrates the diagnostic difficulties that often arise, especially when the clinical and imaging characteristics escape those usually described and when other clinical findings appear as confounding factors. A 17-year-old female was initially admitted to the emergency department (ED) with a holocranial headache, gait imbalance, and vomiting. A brain magnetic resonance imaging (MRI) was done and a rounded lesion was detected in the left cerebellar hemisphere, hypointense in T1 and hyperintense in T2, with annular contrast enhancement. Several hypotheses for diagnosis were made, and the patient was subjected to several therapies, with periods of remission of symptoms interleaved with periods of worsening. After imaging suggestive of hemangioblastoma on routine brain MRI, the tumor was excised surgically and the histopathology confirmed the diagnosis. In the control brain MRI exams performed six and 24 months after surgery, no evidence of tumor recurrence was detected, and the patient remained asymptomatic. In conclusion, although these are rare neoplasms, it is essential to always consider hemangioblastomas in the differential diagnosis of cases with compatible clinical and radiological findings. A wrong or late diagnosis may lead to the use of unnecessary and harmful therapies as well as the appearance of potentially preventable complications if these tumors are handled correctly and timely.

## Introduction

The term “hemangioblastoma,” originally introduced by Cushing and Bailey in 1928, describes a slow-growing, highly vascularized benign neoplasm that typically affects the central nervous system (CNS) [1]. These tumors account for approximately 1-2.5% of all intracranial tumors and 2-3% of all intramedullary neoplasms [2-5]. They can occur in any area of ​​the CNS, even outside it. The cerebellum is, by far, the most common place for its location [3-5].

In nearly 70-80% of the cases, hemangioblastomas appear as isolated, sporadic lesions. The remaining cases are associated with von Hippel-Lindau disease (VHLD), an inherited disease of autosomal dominant transmission characterized by a predisposition to the occurrence of multiple hemangioblastomas and other visceral lesions that develop throughout life [6]. Essentially considered tumors in adults, they show peaks of incidence in the third and fifth decades of life. They are a little more common in males, with a ratio of 1.3 to 2:1 [7].

Despite their benign growth behavior, these tumors can become important causes of morbidity and mortality, with specific symptoms depending essentially on anatomical location and growth pattern [8,9].

Standard treatment consists of surgical excision of symptomatic tumors when the benefits of the procedure outweigh the surgical risk [5,10-13].

Although considered rare neoplasms, it is important to always consider hemangioblastomas in the differential diagnosis of cases that present with clinically compatible radiology. Misdiagnosis or late diagnosis can lead to the use of unnecessary therapies and, eventually, harm to the patient, as well as to the emergence of potentially avoidable complications if handled correctly and in a timely manner. The clinical case presented here, referring to a cerebellar hemangioblastoma, illustrates well the diagnostic difficulties that often arise associated with this type of neoplasm.

## Case presentation

A 17-year-old female, with no medical problems, presented in the emergency department (ED) with holocranial headache, unbalanced gait, rotational vertigo, and vomiting with four days of evolution. On examination, diplopia to left lateralization of the gaze was highlighted, nystagmus in the extreme positions of the gaze, gait deviation to the left, a slight fall of the left upper limb in the outstretched arms test, and balance was maintained in the Romberg test, without visual interference. Brain magnetic resonance imaging (MRI) was performed with the administration of gadolinium contrast, which showed a lesion in the left cerebellar hemisphere, hypointense on T1 and hyperintense on T2, with annular contrast uptake. A diagnostic hypothesis of multiple sclerosis was suggested, and therapy with methylprednisolone was performed for five days. Control MRI was performed on the last day of therapy with dimension reduction of the cerebellar lesion.

There was an improvement in the clinical picture as well as a normalization of the neurological exam, with only left gait deviation persisting. In view of the diagnosis, the patient developed a reactive anxiety-depressive condition and began follow-up in a psychiatric consultation. Six months later, she went to the ED because of symptoms similar to the initial ones, with evidence, on neurological examination, of diplopia in the combined gaze to the left and deviation to the left during gait. A new MRI was performed, showing a lesion in the left cerebellar hemisphere with characteristics similar to the initial one. Remedicated with methylprednisolone for three days, but with no improvement during hospitalization. Somatosensory, visual and auditory evoked potentials without alterations, and lumbar puncture without oligoclonal bands and with negative serology. Despite the images obtained on MRI, the hypothesis of demyelinating disease was discarded, and the possibility of sequelae of cerebellar ischemic stroke was assumed. In view of this hypothesis, an etiological study of stroke in young people was carried out, which showed positive homozygosity for the 4G allelic variant of the plasminogen activator inhibitor-1 gene (PAI-1) and exuberant aneurysm of the interauricular septum with permeable foramen ovale with the passage of stirred serum in copious amounts. The patient underwent surgical closure of the interatrial communication with autologous pericardium approximately 16 months after the initial clinical condition. Medicated for ambulatory care with antiplatelet and oral contraceptive suspension. There were no intercurrences for two years after surgery, except for an episode of thrombosis of the left common femoral vein, which was started with oral hypocoagulation with warfarin, which was maintained for six months.

**Figure 1:**
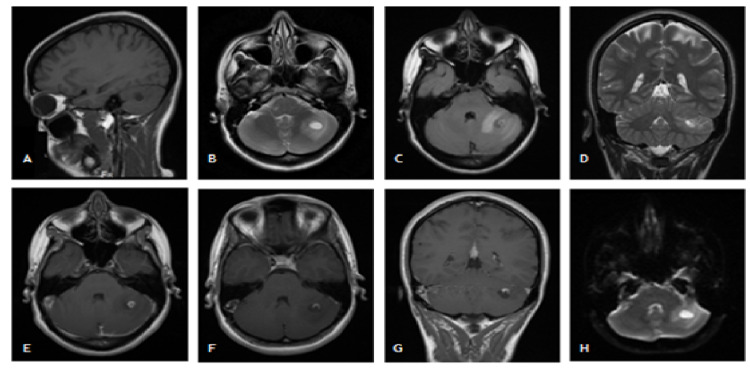
Encephalic MRI showing imaging compatible with left cerebellar hemisphere hemangioblastoma. Rounded lesion, hypointense on T1 and hyperintense on T2, with thick signal reinforcement after gadolinium, approximately 7 mm in diameter, located in the left cerebellar hemisphere; inferiorly to the “ring” lesion, a cyst measuring approximately 12.5 mm in diameter, whose interior weights homogeneously like cerebospinal fluid (CSF); larger area of ​​surrounding edema compared to previous examinations. T1-weighted sagittal plane (A), T2-weighted axial plane (B), T2-weighted axial fluid-attenuated inversion recovery (FLAIR) plane (C), T2-weighted coronal plane (D), T1-weighted axial plane after intravenous administration of gadolinium contrast (E) and (F), T1-weighted coronal plane after intravenous administration of gadolinium (G), axial plane diffusion imaging (H). MRI: magnetic resonance imaging.

After this period, she develops intense holocranial headache and nausea. On neurological examination, oscillations to maintain the orthostatic posture without enlargement of the base, gait with lateral deviations to the left, and fine tremors in the left upper limb on the finger-nose test. A brain MRI was done, which again showed the presence of abnormal blood vessels in the vicinity of the nodular lesion (no evidence of this in the arterial phase). After discussing the case with neuroradiology, the imaging characteristics of the lesion made it possible to establish a differential diagnosis between tuberculoma, primary or secondary tumoral lesion and arteriovenous malformation. It should be noted that the interference of a generalized anxious component in the clinical picture was also considered. A full analytical and microbiological studies of the blood, urine, and cerebrospinal fluid were done and we found a 20 mm positive Mantoux test. Despite the positive Mantoux test, the stability of the lesion dimensions over approximately four years, as well as the presence of abnormal circulation in the periphery, were not suggestive of tuberculosis. It is also important to mention the absence of contacts with people with active infection and an unaltered chest x-ray. However, after discussing the case with infectiology, medication with rifampicin, isoniazid, and pyridoxine was started, but suspended after six months due to stability of the lesion documented in a new control brain MRI. Due to diagnostic uncertainty, the patient was evaluated in a neurosurgery consultation six years after the onset of the clinical picture. Control MRI was performed and the radiological aspects, after comparison with previous exams, were suggestive of hemangioblastoma (Figure 1).

The patient underwent elective tumor excision surgery through a left paramedian suboccipital craniotomy with total macroscopic tumor removal. The neuropathological report confirmed that the excised tissue was a benign angiomatous neoplastic lesion, corresponding to a cerebellar hemangioblastoma. It should be noted that there is no family history of similar lesions in any region of the CNS, but a detailed imaging study was carried out, including MRI of the cervical and dorsal spine, abdominal and pelvic CT, as well as retinal angiography, to exclude VHLD; none of the exams revealed lesions with pathological significance. The patient refused to carry out a genetic study to investigate the mutation in the von-Hippel Lindau (gVHL) gene. Control encephalic MRIs were also performed at six and 24 months after surgery, which showed no evidence of tumor recurrence or new cranial lesions.

## Discussion

Although considered rare neoplasms, accounting for only 1-2.5% of all intracranial neoplasms, hemangioblastomas comprise about 7-12% of posterior cranial fossa tumors, constituting the most common type of primary intra-axial tumor of those that form in this location in adults [4]. Thus, it is essential to always consider hemangioblastomas in the differential diagnosis of cases that present compatible clinical and radiological findings. According to the literature, the average duration of symptoms associated with hemangioblastoma until diagnosis is about 13 months [7].

It is known that approximately 95% of infratentorial hemangioblastomas are located in the cerebellum, with the cerebellar hemispheres being more commonly affected than the vermis [10]. Posterior fossa hemangioblastomas are predominantly manifested by symptoms of intracranial hypertension caused by the mass effect exerted by the tumor, by peritumoral cysts and associated edema, as well as by blocking the cerebrospinal fluid circulation pathways by obstruction of the fourth ventricle [7,10,14]. The latter situation can lead to the occurrence of hydrocephalus with rapid clinical decompensation [2,7]. Headache constitutes the most common symptom at presentation, being present in 95% of the cases. After this, nausea, vomiting, vertigo, dizziness, diplopia, nystagmus, and gait disturbances are the clinical manifestations most commonly presented by patients [7,10]. The neurological examination may be normal or show characteristic signs of a cerebellar syndrome. On funduscopy, papilloedema is often found [10]. MRI is extremely sensitive and specific in the early diagnosis of hemangioblastomas, especially when associated with intravenous administration of gadolinium contrast; it constitutes the gold standard exam for the diagnosis and follow-up of these lesions. Despite its sensitivity, false negative results can occur with lesions <5 mm and with late imaging due to early enhancement of lesions after contrast administration [7,10].

In this case, the diagnosis was probably delayed due to several factors: the patient had symptoms compatible with the location of the lesion in the left cerebellar hemisphere, but some symptoms emerged that could not be explained by the lesion, but rather by an occasional anxious-depressive condition; the initial radiological features, seen on encephalic MRI, were not suggestive of hemangioblastoma; in addition, throughout the etiological investigation process, new clinical findings compatible with other diagnostic hypotheses emerged. It should be noted that the symptomatology of hemangioblastoma is not specific and depends on the anatomical location of the tumor, its growth pattern, the compressive effect it causes on adjacent structures, as well as the surrounding edema, which can make the diagnosis even more difficult [9].

Although the clinical outcome was favorable after the tumor excision surgery, fatal complications could have occurred, namely, massive hemorrhage in the posterior cranial fossa. Although the rich vascularization and asymptomatic and microscopic intratumoral hemorrhages are characteristic features of this type of tumor, massive hemorrhage is a very uncommon event (0.0024 per patient per year). It may, however, be the initial manifestation [14].

After the definitive pathological diagnosis of cerebellar hemangioblastoma, the patient underwent an extensive clinical study in order to exclude the possibility of VHLD. However, a genetic study was not carried out to detect the germline mutation associated with gVHL, responsible for the disease. This was due to the patient's refusal to perform the study. Apparently, the patient's hemangioblastoma is sporadic as there is no family history of similar lesions and no additional lesions were detected in the studies performed, but the diagnosis of VHLD cannot be definitively ruled out. On the one hand, the patient was 17 years old when the symptoms began, a very early age for it to be a sporadic lesion, whose age at diagnosis is usually between 30 and 65 years old [8]; on the other hand, the diagnosis of VHLD is subsequently confirmed in about 25-30% of cases of apparently sporadic CNS hemangioblastomas [9].

Special attention should be paid to the underdiagnosis of VHLD in patients with apparently isolated lesions and no evidence of family history, with some authors recommending genetic screening in all patients diagnosed with CNS hemangioblastoma, even if apparently sporadic [15]. It would be important that this patient undergo a genetic study so that, if positive, she is regularly monitored for early diagnosis of new lesions and appropriate intervention at the right time, avoiding predictable complications associated with the lesions.

Patients have a very favorable long-term prognosis after complete surgical resection of the tumor. However, these neoplasms have recurrence rates of up to 27% with a symptom-free interval of, on average, five years [7]. Thus, the patient's follow-up is necessary, as its duration depends on several clinicopathological factors [7,12,13]. No evidence-based suggestions for follow-up after removal of a sporadic CNS hemangioblastoma are found in the literature, although some authors suggest performing MRI of the involved neural axis at six and 12-24 months after the excisional surgery. However, follow-up modalities must be individualized and based on clinical indications [15]. In control MRIs, performed six and 24 months after surgery, no evidence of tumor recurrence was detected. However, it is important that the patient is made aware of the possibility of relapse as well as the possibility of VHLD and, therefore, seek medical assistance whenever neurological symptoms arise again.

## Conclusions

The clinical case presented, referring to a cerebellar hemangioblastoma, illustrates well the diagnostic difficulties that often arise when the clinical and imaging characteristics are beyond those usually described and when other individual characteristics and clinical findings that can act as confounding factors are present. This case involved approximately six years of simultaneous follow-up by several medical specialties and in different hospitals, from the onset of symptoms to the appropriate diagnosis and treatment of the cerebellar hemangioblastoma. During this period of time, several diagnostic hypotheses were raised for the lesion found in the cerebellum without, however, ever having considered the hypothesis of hemangioblastoma, and the patient, still very young, was subjected to the use of unnecessary and, eventually, stigmatizing therapies. Misdiagnosis or late diagnosis can lead to the use of unnecessary therapies and, eventually, harm to the patient, as well as to the emergence of potentially avoidable complications if handled correctly and in a timely manner. Thus, although these are rare neoplasms, it is essential to always consider hemangioblastomas in the differential diagnosis of cases that present compatible clinical and radiological findings.
